# Cerebral Venous Thrombosis Due to Diabetic Ketoacidosis in a Patient Newly Diagnosed With Diabetes: A Case Report

**DOI:** 10.7759/cureus.79313

**Published:** 2025-02-19

**Authors:** Mohamed Hanana, Ouassima Bernichi, Wiam Ftouh, Fatima Aziouaz, Mariem Benkacem

**Affiliations:** 1 Department of Endocrinology, Diabetes and Metabolic Diseases, Mohammed VI University Hospital, Tangier, MAR

**Keywords:** cerebral venous thrombosis, diabetes, diabetic ketoacidosis, hyperglycemic emergency, ketoacidosis complications, metabolic acidosis

## Abstract

Diabetic ketoacidosis (DKA) is a severe and life-threatening condition associated with diabetes mellitus, characterized by profound metabolic acidosis, ketosis, elevated blood glucose levels, and disturbances in electrolytes. While these are the typical features, DKA can also lead to rare but potentially life-threatening complications such as cerebral venous thrombosis. The hypercoagulable state associated with DKA, exacerbated by dehydration and diabetes itself, promotes coagulation. We present the case of a 33-year-old man with no significant medical history, who was admitted with acute symptoms of lethargy, vomiting, and chills. He was diagnosed with DKA and managed with intravenous insulin, fluid resuscitation, and electrolyte correction. During recovery, he developed meningeal symptoms, and imaging revealed a cerebral venous thrombosis. Anticoagulation therapy led to significant clinical improvement. This case highlights the importance of recognizing rare thrombotic complications in DKA such as cerebral thrombosis. It highlights the need for prompt management and further investigation into the role of anticoagulation therapy in DKA.

## Introduction

DKA is a life-threatening metabolic disorder characterized by acidosis and dehydration due to insulin deficiency, primarily affecting individuals with type 1 diabetes mellitus but occasionally occurring in those with type 2 diabetes [1]. A well-recognized complication of DKA is a hypercoagulable state, which increases the risk of arterial and venous thromboembolic events, including cerebral venous thrombosis [2]. 

Cerebral venous thrombosis is a rare but serious neurological complication that can present with non-specific symptoms such as headache, seizures, focal neurological deficits, or altered mental status, often mimicking other conditions like meningitis, stroke, or encephalopathy [3]. Diagnosis relies on neuroimaging, particularly magnetic resonance imaging (MRI), to confirm venous sinus occlusion. Management typically includes hydration, insulin therapy, and anticoagulation, with close monitoring for complications such as hemorrhagic transformation or raised intracranial pressure [4]. This report highlights cerebral venous thrombosis as a rare but significant manifestation of DKA in a young adult with newly diagnosed type 1 diabetes, underscoring the importance of early recognition and intervention to prevent severe neurological outcomes.

## Case presentation

A 33-year-old male patient was admitted to the emergency department with a four-hour history of vomiting, lethargy, and chills. The patient had no significant medical history. At admission, he was lethargic, pale, and diaphoretic, with drowsiness. His conscious state, evaluated using the Glasgow Coma Scale (GCS), was 14/15 (spontaneous eye-opening: 4, confused verbal responses: 4, and obeys commands for motor response: 6). He was tachycardic (heart rate: 120 beats per minute), with hyperventilation (respiratory rate: 30 breaths per minute). His family reported that this condition appeared suddenly without an evident cause. The patient had no history of head trauma or drug or alcohol use and had been in good health until that day.

Initial investigations revealed a significantly elevated blood glucose level of 520 mg/dL (measured by a glucometer), and a urinary evaluation using a urine dipstick revealed ketosis at 3+. These findings, combined with symptoms of dehydration, rapid breathing, and a fruity odor on the breath, were consistent with the diagnosis of DKA. As a result, an insulin-based management protocol was initiated along with fluid and electrolyte replacement therapy. This included intravenous fluids at a normal maintenance rate (0.45% saline with 5% dextrose) and intravenous insulin (at 0.1 units/kg/hour).

Blood samples were also taken to assess electrolyte balance and the degree of ketosis. Laboratory results of the initial samples showed a significantly elevated blood glucose level of 500 mg/dL (normal: 70-100 mg/dL), marked metabolic acidosis with a pH of 7.1 (normal: 7.35-7.45), and bicarbonate levels of 12 mEq/L (normal: 22-28 mEq/L). The anion gap was increased to 18 mEq/L (normal: 8-12 mEq/L), indicating ketoacidosis. Potassium was elevated at 5.5 mEq/L (normal: 3.5-5.0 mEq/L) but was expected to drop with treatment. Sodium was low at 130 mEq/L (normal: 135-145 mEq/L). Renal investigations revealed evidence of hypoperfusion, including elevated creatinine levels of 2.0 mg/dL (normal: 0.6-1.2 mg/dL) and a blood urea (BU) level of 30 mg/dL (normal: 7-20 mg/dL). Chloride was slightly reduced at 95 mEq/L (normal: 98-106 mEq/L). C-reactive protein (CRP) was mildly elevated at 10 mg/L (reference range: <3 mg/L), which, along with the absence of a clinically evident infection, ruled out a significant infectious cause for decompensation. Other parameters, including a complete blood count and hepatic function tests, were within normal limits with no notable abnormalities.

The patient’s condition improved rapidly. On re-evaluation, he was fully conscious with a GCS of 15/15 (spontaneous eye-opening: 4, appropriate verbal responses: 5, and obeys commands for motor response: 6). He was hemodynamically stable, had a normal respiratory rate, and blood glucose level dropped to 160 mg/dL. The management of DKA was successful, and the patient was started on subcutaneous insulin therapy.

By the second day, the clinical course was complicated by the development of meningeal syndrome, mainly neck stiffness. Infectious meningitis was suspected, given the immunocompromised state associated with diabetes. A lumbar puncture was performed, along with empirical antibiotics administration. However, cerebrospinal fluid (CSF) analysis showed normal results, with no white blood cells, a few red blood cells, and no identifiable microorganisms.

**Figure 1:**
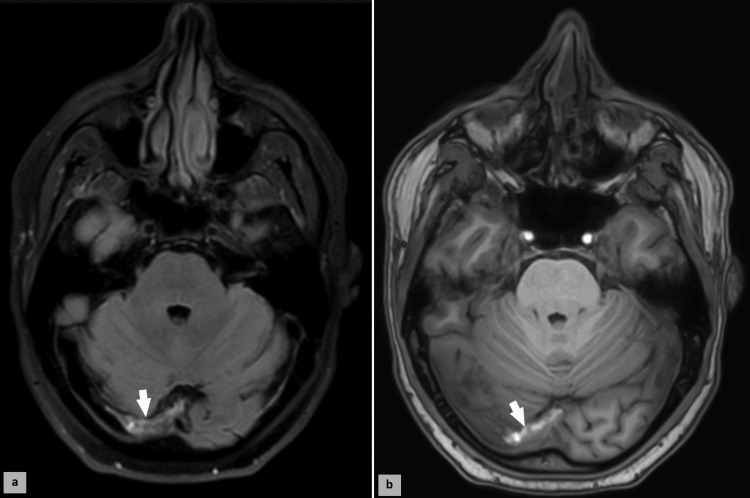
MRI of the brain (axial views): (a) T1-weighted image demonstrating left lateral venous thrombosis in the posterior fossa (arrow), and (b) T1-weighted contrast-enhanced image depicting the corresponding thrombus with hyperintense signal (arrow).

Due to the persistence of meningeal symptoms, an MRI was performed, revealing a thrombosis of the right lateral venous sinus (Figure 1). The patient was started on anticoagulation therapy with heparin and showed good clinical and radiological improvement. A complete prothrombotic workup, including tests for antiphospholipid antibodies, protein C and S levels, antithrombin III levels, and factor V Leiden mutation, was performed, but all results were normal. The sinus thrombosis was attributed to the hypercoagulable state induced by DKA.

The patient was discharged in stable condition on a long-term insulin therapy regimen and enrolled in a diabetes education program. He was also provided with instructions for regular follow-up and surveillance.

## Discussion

DKA is the most frequent acute hyperglycemic emergency encountered in individuals with diabetes, particularly those with type 1 diabetes [3]. This condition arises from an absolute insulin deficiency caused by autoimmune destruction of pancreatic beta cells. The resultant lack of insulin triggers a cascade of metabolic derangements, including hyperglycemia, dehydration, metabolic acidosis, and ketosis, which is characterized by elevated ketone levels in the blood or urine (serum ketone concentration >3.0 mmol/L) [4]. While DKA is predominantly associated with type 1 diabetes, it is infrequently reported in individuals with type 2 diabetes. DKA is associated with significant morbidity [4]. In developing countries, mortality rates among children can reach up to 13%, with cerebral injuries and edema being the primary contributors in this population [5].

The severe metabolic acidosis observed in DKA is primarily due to enhanced lipolysis, where fatty acids are metabolized into ketones in the liver. Simultaneously, hyperglycemia leads to osmotic diuresis, which causes significant fluid loss and dehydration [6]. The hypercoagulable state associated with DKA increases the risk of vein thrombosis, pulmonary embolism, and stroke [4]. Among the serious complications of DKA, cerebrovascular accidents stand out as potentially life-threatening [7]. Cerebral complications associated with DKA, such as cerebral edema, thrombosis, hemorrhage, and infarction, are rare, occurring in approximately 0.9% of pediatric cases and even less frequently in adults [8].

Studies have demonstrated that DKA induces a reduction in protein C activity and free protein S levels, accompanied by a significant elevation in von Willebrand factor (vWF) antigen and activity [7]. Platelet activation has also been observed during episodes of DKA, further contributing to a prothrombotic state [2]. Endothelial damage and an amplified inflammatory response have been implicated in this process as well [9]. Additional factors, including hyperosmolarity, hemoconcentration, and dehydration, exacerbate the hypercoagulable state [10]. Dehydration increases blood viscosity, leading to higher concentrations of coagulation proteins and platelets, further heightening thrombotic risk. These changes foster a prothrombotic environment, raising the likelihood of both venous and arterial thrombosis [1].

In our case, the patient exhibited moderate DKA, characterized by altered mental status and biochemical abnormalities consistent with the American Diabetes Association's diagnostic criteria for DKA in adults. These included glucose >250 mg/dL, venous pH of 7.24-7.0, bicarbonate levels between 10-15 mmol/L, positive urine or serum ketones, an anion gap >12, and drowsiness [4,11].

Management of DKA involves prompt fluid resuscitation, insulin therapy, potassium supplementation, and treatment of the underlying precipitating factor [12]. These interventions aim to correct acidemia, restore circulatory volume, and normalize blood glucose levels [11]. With appropriate therapy, metabolic disturbances and acidosis typically resolve within 24 hours [13]. Infections are common precipitating factors for DKA. Other triggers include acute illnesses, malfunction of insulin delivery devices, and non-adherence to prescribed insulin therapy in treated diabetic patients [4,14], Therefore, a comprehensive evaluation is advised to identify any underlying factors contributing to decompensation.

This case highlights the thromboembolic risk associated with DKA. Our patient developed a thromboembolic event, which involves factors such as dehydration, platelet activation, and endothelial damage. This case underscores the need for further research to better understand the mechanisms driving thrombosis in DKA and to identify why some patients are more prone to thromboembolic events than others.

## Conclusions

The occurrence of cerebral venous thrombosis in DKA, although rare, highlights the critical need for vigilant monitoring of unusual complications in patients with diabetes. This case underscores the importance of early recognition and intervention in DKA management. Additionally, it emphasizes the need for further research into the mechanisms behind thromboembolic events in DKA to better identify patients at increased risk and improve outcomes. Timely management and comprehensive evaluation remain key to preventing and addressing the complex complications associated with this life-threatening condition.
